# Supplementary figures and images for: Deep self-supervised learning for biosynthetic gene cluster detection and product classification
Source: PLoS Comput Biol. 2023 May 23;19(5):e1011162. doi: 10.1371/journal.pcbi.1011162 (PMC10241353; doi:10.1371/journal.pcbi.1011162)

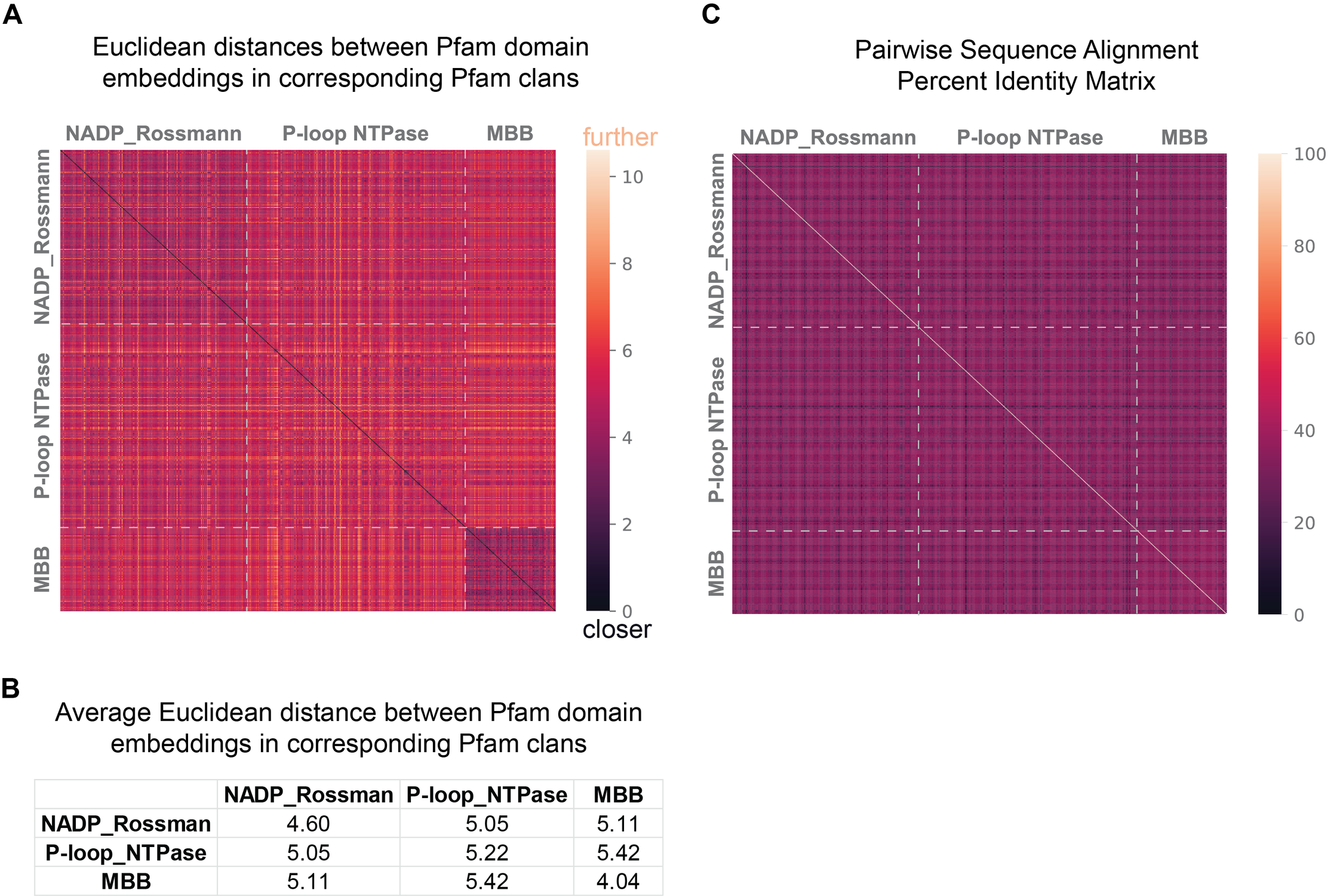

Supplement: S1 Fig — (a) Heatmap of Euclidean distances between domain embeddings in the following Pfam clans: NADP_Rossmann, P-loop NTPase, and MBB. (b) Average Euclidean distance between Pfam domain embeddings in the aforementioned Pfam clans (c) Pairwise sequence alignment percent identity matrix between Pfam clans. (TIF) [file pcbi.1011162.s001.tif]
